# Exploring barriers and enablers for scaling up a community‐based grain bank intervention for improved infant and young child feeding in Ethiopia: A qualitative process evaluation

**DOI:** 10.1111/mcn.12551

**Published:** 2017-10-24

**Authors:** Binta Sako, Joanne N. Leerlooijer, Azeb Lelisa, Abebe Hailemariam, Inge D. Brouwer, Amal Tucker Brown, Saskia J. M. Osendarp

**Affiliations:** ^1^ Independent Consultant Brussels Belgium; ^2^ University of Applied Sciences Utrecht The Netherlands; ^3^ Nutrition International Addis Ababa Ethiopia; ^4^ UNICEF Addis Ababa Ethiopia; ^5^ Wageningen University Wageningen The Netherlands; ^6^ Nutrition International Ottawa Ontario Canada

**Keywords:** complementary feeding, Ethiopia, infant and child, nutrition, nutritional interventions, process evaluation, scaling‐up

## Abstract

Child malnutrition remains high in Ethiopia, and inadequate complementary feeding is a contributing factor. In this context, a community‐based intervention was designed to provide locally made complementary food for children 6–23 months, using a bartering system, in four Ethiopian regions. After a pilot phase, the intervention was scaled up from 8 to 180 localities. We conducted a process evaluation to determine enablers and barriers for the scaling up of this intervention. Eight study sites were selected to perform 52 key informant interviews and 31 focus group discussions with purposely selected informants. For analysis, we used a framework describing six elements of successful scaling up: socio‐political context, attributes of the intervention, attributes of the implementers, appropriate delivery strategy, the adopting community, and use of research to inform the scale‐up process. A strong political will, alignment of the intervention with national priorities, and integration with the health care system were instrumental in the scaling up. The participatory approach in decision‐making reinforced ownership at community level, and training about complementary feeding motivated mothers and women's groups to participate. However, the management of the complex intervention, limited human resources, and lack of incentives for female volunteers proved challenging. In the bartering model, the barter rate was accepted, but the bartering was hindered by unavailability of cereals and limited financial and material resources to contribute, threatening the project's sustainability. Scaling up strategies for nutrition interventions require sufficient time, thorough planning, and assessment of the community's capacity to contribute human, financial, and material resources.

## 1 INTRODUCTION

Ethiopia is among the countries most affected by child malnutrition, with high rates of stunted growth affecting more greatly the rural poor. Even though rates are declining, 38% of Ethiopian children under 5 remain stunted (Central Statistical Agency, CSA, & ICF, 2016). Most children do not fulfil requirements for adequate feeding: only half (56%) of children 6–8 months received complementary food, whereas 7% received a minimum adequate diet, consisting of minimum dietary diversity and age‐dependent minimum feeding frequency (CSA & ICF, 2016). Adequate infant and young child feeding (IYCF) is one of the most important strategies to address the causes of childhood undernutrition (Bhutta et al., 2013), and interventions that combine education and food provision in areas with widespread food insecurity are particularly known to be effective (Bhandari et al., 2004; Dewey & Adu‐Afarwuah, 2008; Roy et al., 2007). Integrating such nutrition interventions into effective, large‐scale development programs can further accelerate impact on mortality. It is estimated that proven nutrition interventions, when implemented at a coverage of 90%, could reduce the under‐5 child mortality rate by 15% in the 34 countries with the highest burden of malnutrition (Bhutta et al., 2013).

In the past decade, there has been increasing attention for scaling up of nutrition interventions (Bhutta et al., 2013; Gillespie, Haddad, Mannar, Menon, & Nisbett, 2013; International Food Policy Reaserch Institute, 2012). Despite known benefits, few efficacious IYCF interventions are scaled up due to organizational and resource constraints. The Alive and Thrive initiative, however, has scaled up programs globally to prevent stunting (Piwoz, Baker, & Frongillo, 2013). A comprehensive review documenting these experiences and lessons learned showed that the lack of scale‐up implementation of IYCF programs could be attributed to a combination of reasons: a lack of scale strategies and resources to support them, incomplete understanding of economic and cultural barriers, and incorrect assumptions about determinants of poor feeding practices, such as assuming that food insecurity or poverty is the underlying cause of poor complementary feeding (Piwoz et al., 2013). One of the few successfully scaled‐up IYCF interventions in Bangladesh was designed along these principles and identified as success factors for achieving scale up: streamlining of tools and strategies, government branding, phased expansion through partners with community‐based platforms, and nationwide mainstreaming through multiple non‐governmental and government programs (Sanghvi et al., 2016). However, there is still a lack of implementation research that has evaluated programs systematically to better understand the processes involved in scaling up (Menon et al., 2014; Robert et al., 2006). Various frameworks and models provide guidance to plan and evaluate scaling up of health interventions (Milat, Bauman, & Redman, 2015). A framework by Yamey (2011) was developed in a context of global health interventions and identified six elements for successful scale up: supportive socio‐political context, attributes of the intervention, attributes of the implementers, delivery strategy, adopting community, and research, monitoring, and evaluation.

In 2010, UNICEF, in partnership with the Food and Agricultural Organization of United Nations, Addis Ababa University, and regional universities, launched a pilot project using an innovative approach to improve access to quality complementary food in four regions: Amhara, Tigray, Southern Nations Nationalities and Peoples Region (SNNPR), and Oromia (Watanabe, 2013). The innovation consisted of the participation of beneficiary mothers in the production of complementary food made of local ingredients in so‐called “grain banks” and a subsidized bartering system. The results were promising and prompted interest in scaling up the intervention, commonly referred to as the “Grain Bank project,” to reach a larger number of beneficiaries. Scaling up increased the scope of intervention from eight rural kebeles^1^ in the initial pilot, supervised by four regional universities, to 180 rural kebeles, implemented by two local non‐governmental organizations (NGOs) in the same four regions. The scaled‐up intervention model included in addition a reinforcement of the participatory approach of the intervention by involving the community more in the daily management decisions, an increased integration with community nutrition and agriculture programs, training and capacity building of community level staff to support activities, and a behaviour change strategy to increase demand and mobilize the community.

Guided by the Yamey (2011) framework, the purpose of this study was to identify enablers and barriers for successful scaling up of the Grain Bank project. We therefore conducted a qualitative case study, interviewing key informants and community groups involved in the project and representing the six thematic areas of the framework, on their experiences with the project.

**Key messages**

- Of all different aspects of an integrated community‐based nutrition project, mothers of children 6–23 months expressed they most valued learning and adopting new feeding practices to improve their children's diet.
- National and regional stakeholders of the project emphasized that despite government support and local ownership, scaling up a complex Infant and Young Child Feeding intervention requires time to achieve full engagement at community level, and thorough planning and concerted efforts to mitigate health system constraints.
- A strong political will, alignment of the intervention with national priorities, and integration in the health care system were instrumental in scaling up the nutrition project.

## 2 METHODS

### 2.1 Description of the intervention

The 2‐year Grain Bank project combined nutrition education with provision of quality affordable complementary food to children 6–23 months. In selected kebeles, a grain bank was constructed, and a group of 25 women was selected to produce complementary food following a predefined recipe^2^ of local legumes and cereals. Legumes included field peas, chickpeas, broad beans, and kidney beans. Cereals included maize, teff, wheat, and sorghum. The women used traditional methods to wash, roast, germinate, and dehull the grains, improving the nutritious properties and palatability of the flour.

The flour was distributed monthly with the support of health extension workers (HEWs) to families with children 6–23 months in the community. It was recognized that the proposed complementary food mix lacked in fat and various micronutrients (Addis Ababa University, 2010). Consequently, during monthly distribution, caregivers were advised to include fat, animal products, and additional fruits and vegetables when preparing the porridge made with the grain bank flour.

**Figure 1:**
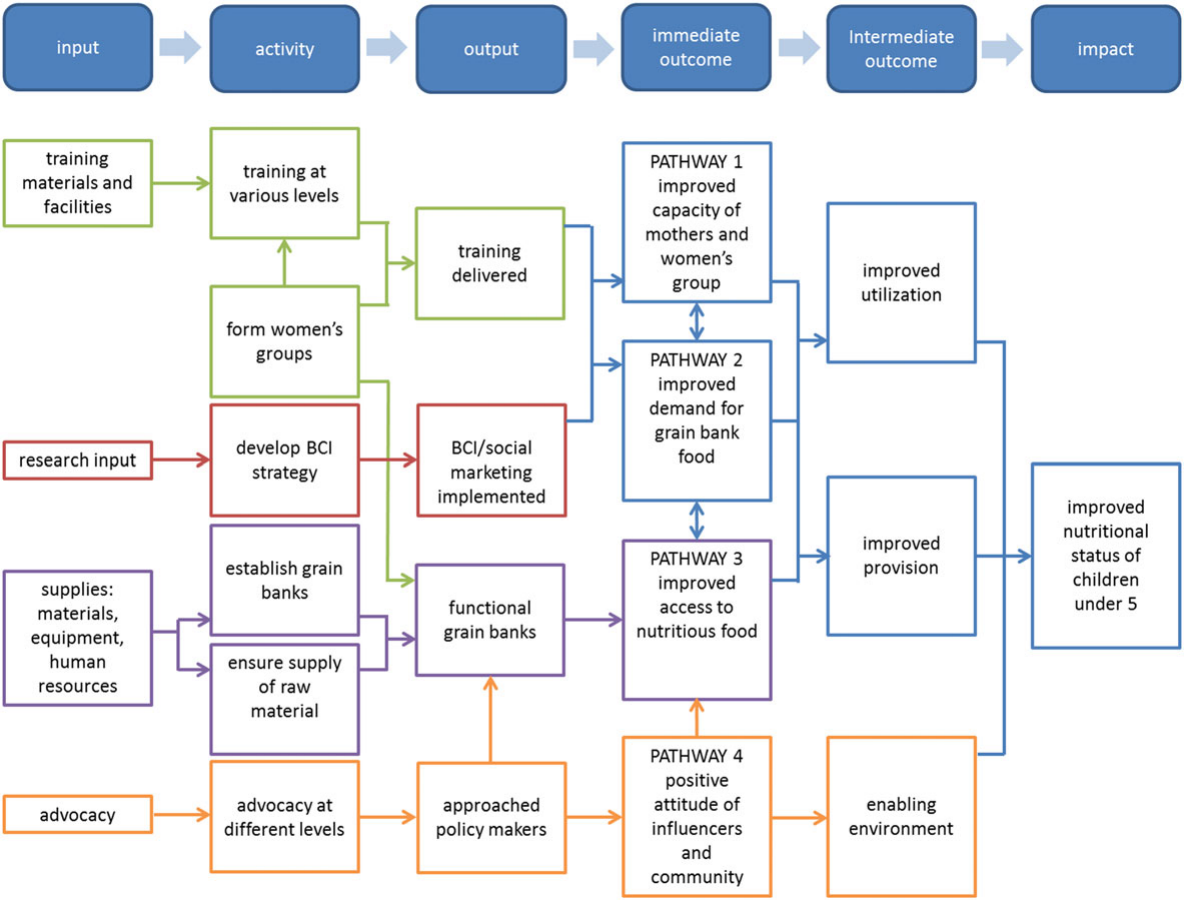
Logic model of the Grain Bank project. BCI = Behaviour Change Intervention

Reviews of the pilot project showed wide acceptance of bartering (Roche, Sako, Osendarp, Adish, & Tolossa, 2017; Watanabe, 2013), whereas the purchasing power of rural women was known to be limited. Therefore, the Grain Bank project maintained the bartering system for the scale up. In this mode of exchange, benefitting mothers contributed cereals available at the household and received the grain bank flour per a barter rate of 2:3.^3^ This rate could be negotiated and was decided at community level by community leaders and representatives. The community was expected to provide firewood, water, and milling expenses, whereas the project donors provided the stock of legumes and cereals required, construction cost, and initial equipment. The logic model of the intervention is illustrated in Figure 1 showing the pathways that were expected to lead to improved nutritional status of children under 5.

During the course of the Grain Bank project, 4,500 women were trained to produce local grain bank complementary food, and an estimated 36,000 children 6–23 months were benefitting from the project.

### 2.2 Approach to the evaluation

Milat et al. (2015) performed a literature review and described eight frameworks and models for scaling up health interventions from which they identified key success factors and barriers for scaling up. One of the eight frameworks was developed by Yamey (2011) in a context of global health interventions and among others informed by the “Diffusion of Innovations theory” of Rogers (1995).

This framework identifies six elements for successful scale up: (a) socio‐political context, (b) attributes of the intervention, (c) attributes of the implementers, (d) delivery strategy, (e) adopting community, and (f) research and monitoring and evaluation (M&E). We chose this framework as a basis to investigate the Grain Bank project because of its comprehensiveness in aspects of scaling up that were relevant to our Grain Bank project.

### 2.3 Selection of study sites

The intervention established one grain bank in each kebele. We used coverage data reported in the monitoring reports, indicating the number of children 6–23 months that was offered the complementary feeding intervention monthly, as a performance indicator. The grain banks were ranked from the highest to lowest average coverage per region and per woreda.^4^ In each of the four regions, we selected one high‐ and one low‐performing grain bank out of 45 grain banks (eight grain banks in eight kebeles in total), each in a different woreda, while considering logistic accessibility. By contrasting the performance levels, the intention was to gain a broader range of experiences and to be able to identify enablers and barriers for successful scale up in each region. All intervention kebeles were also part of the Community‐Based Nutrition Program and benefitted from enhanced nutrition education and services (UNICEF‐Ethiopia, 2013).

### 2.4 Selection of study participants

**Figure 2:**
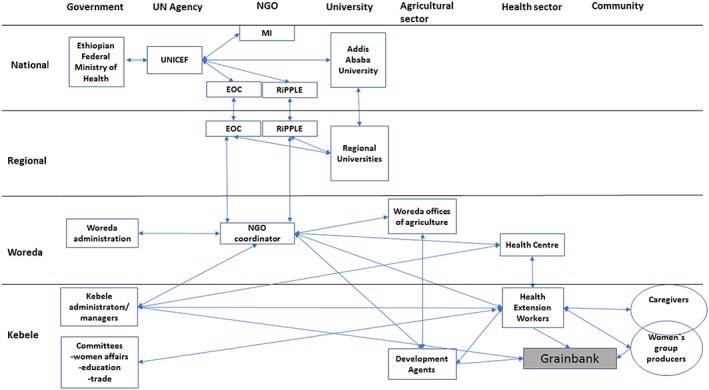
Key stakeholders involved in the Grain Bank project. UN = United Nations; NGO = non‐governmental organizations; UNICEF = United Nations International Children's Emergency Fund; EOC = Ethiopian Orthodox Church; MI = micronutrient initiative

We purposely recruited key informants from the selected kebeles and woredas and stakeholders from national and regional levels to participate and share experiences and opinions on the project. Figure 2 shows the key stakeholders involved in the project and depicts the complexity of the intervention: a multitude of stakeholders from different institutions working at different levels and their interrelations. Key informants were selected based on their specific role in the project and community groups for their involvement as actors or influencers in the project. Fifty‐two key informant interviews (KIIs) were conducted. These also included six interviews with senior‐level stakeholders of donor agencies, implementing NGOs, and universities, which were held in Addis Ababa, prior to data collection at woreda and kebele level (see Table 1). We conducted 31 of the 32 planned focus group discussions (FGDs). Table 2 provides details of participants in KIIs and FDGs at community levels.

**Table 1:** Participant groups in key informant interviews and focus group discussions and topics covered

Methods	Topics
Key informant interviews participants	
Senior officials from UNICEF, micronutrient initiative, RiPPLE^1^, EOC^2^, and Addis Ababa University	Description of intervention, successes and main challenges, roles and responsibilities, perception of sustainability
Kebele managers/administrators	Roles and responsibilities in the project, perceptions of sustainability, acceptability, and support of the project by the community
Health extension workers	Description of Grain Bank project modalities, management features of the project, perceptions of sustainability, determinants of success, collaboration and coordination of activities, integration, coverage, adhesion and consumption, acceptability
Woreda and kebele health workers	Integration with health services, roles and responsibilities, coverage, adherence and consumption, perception of sustainability Collaboration with Health Development Army and health extension workers
Agriculture workers/development agents	Integration between agriculture interventions and the Grain Bank project, perception of sustainability
Women's group (Health Development Army) leader/representative	Experiences with processes of preparing, distributing complementary food, decision‐making, quality assurance, perceptions of sustainability, barriers and enablers to participation
Junior nutrition officer/NGO coordinator	Description of Grain Bank project modalities, management features of the project, perceptions of sustainability, determinants of success, collaboration and coordination of activities, integration, coverage, adhesion and consumption, acceptability
Focus group discussions participants	
Caregivers/mothers of children 6–23 months	Acceptability and preferences, barriers and enablers of the Grain Bank project
Influencers: fathers	Community involvement and support to caregivers for the intervention, opinions on the Grain Bank project
Women's group/Health Development Army; women's affairs representative	Community involvement and support, roles and responsibilities in the programme, opinions on the Grain Bank project
Review of project documents	Tranche and annual project reports, coverage data

*Note*. RiPPLE = Research‐inspired Policy and Practice Learning in Ethiopia; EOC = Ethiopian Orthodox Church; UNICEF = United Nations International Children's Emergency Fund; NGO = non‐governmental organizations.

1 Local implementing NGO in SNNPR and Oromia.

2 Local implementing NGO in Amhara and Tigray.

**Table 2:** Details of sampling for focus group discussions and key informant interviews at woreda and kebele level per region

Region	SNNPR	Oromia	Tigray	Amhara
Focus group discussions				
Participant mothers^1^ (no. of groups [*n*, age range])	2 (12, 19–45)	2 (20, 18–38)	2 (15, 20–37)	2 (10, 22–38)
Non‐participant mothers^2^ (no. of groups [*n*, age range])	2 (10, 15–35)	2 (21, 18–40)	2 (15, 20–47)	1 (3, 20–28)
Fathers^3^ (no. of groups [*n*, age range])	2 (11, 24–50)	2 (16, 18–75)	2 (17, 32–65)	2 (11, 20–45)
Health Development Army (no. of groups [*n*, age range])	2 (17, 19–40)	2 (11, 22–55)	2 (11, 20–45)	2 (10, 20–60)
Total (no. of groups [*n*])	8 (50)	8 (68)	8 (58)	7 (34)
Key informant interviews				
Kebele managers/ administrators (*n*)	2	2	2	2
Development agents (*n*)	2	2	2	2
Health extension workers (*n*)	2	2	2	2
Regional NGO coordinator (*n*)	—	—	—	1
Junior nutrition officer/NGO coordinator (*n*)	2	1	1	—
Women's group leader (*n*)	2	2	2	2
Health extension worker supervisor/woreda nutrition officer (*n*)	2	2	2	2
Woreda maternal child health staff (*n*)	—	—	1	—
Total KII (*n*)	12	11	12	11

*Note*. SNNPR = Southern Nations Nationalities and Peoples' Regions; KII = key informant interview; NGO = non‐governmental organizations.

1 Participant mothers are caregivers who participated at least once in the bartering scheme.

2 Non‐participant mothers never participated in the bartering scheme.

3 Fathers include husbands of participating and non‐participating mothers.

### 2.5 Data collection

The research team developed data collection tools specific to each group of respondents, following a review of program documents, the six elements of successful scale up (Yamey, 2011), and interviews with senior‐level stakeholders. We trained experienced data collectors on the data collection tools to ensure quality and consistency. After a pretest, we made minor adaptations to ensure adequacy of the tools. We performed data collection in December 2015 and January 2016 in the four intervention regions. All KIIs and FGDs were audio‐taped and conducted by an interviewer and a transcriber in the respondents' language.

The data collectors informed each participant of the purpose of the study and prior to each KII and FGD. Each participant provided oral or written consent. For FGD participants, data on age, sex, marital status, education, and occupation were also collected. To ensure confidentiality, identity of the respondents and kebele names were known only by the research team. The four regional health bureaus granted approval for the study. Data files are stored safely and will be destroyed after 5 years to comply with international standards.

### 2.6 Data management and analysis

The research team transcribed 20 FGDs and 30 KII verbatim and translated these in English. The team summarized all other interviews (22) and FGDs (11) in English. We coded and analysed transcripts and summaries with Atlas.ti version 7. Coding and analysis followed a combination of deductive and inductive approaches, including the framework developed by Yamey (2011).

## 3 RESULTS

### 3.1 Socio‐political context

The scaling up of the Grain Bank project was aligned with the revised National Nutrition Plan launched in 2013. The National Nutrition Plan objectives and core values comprised multisectorial integration, ownership at community level, providing access to quality affordable complementary food to children 6–23 months, and nutrition education. The intervention also built on the Health Extension Program, a flagship program in Ethiopia, first by choosing sites that were part of the Community‐Based Nutrition Program and second by using the HEWs as frontline actors in the Grain Bank project. Thus, government officials at national, woreda, and kebele levels expressed their support during interviews considering the project part of a national priority and inherent to their duties. A government official expressed his support in these words:

“Considering that key window of opportunity …, mothers, if they get knowledge and skills, can work with their colleagues and prepare the complementary food and feed the child. Such a pilot should be scaled‐up and every kebele, every household should participate.”*—*National stakeholder.

### 3.2 Attributes of the intervention

#### 3.2.1 The bartering model

The intervention consisted of a kebele‐based bartering system where mothers of children 6–23 months exchanged a single raw cereal for a flour made of various legumes and cereals according to a barter rate. The barter rates varied per site from the initial rate of 2:3 (mothers gave two units of any grain, usually cereals, and received three units of complementary foods), to a rate of 3.5:3. Moving away from a favourable exchange rate was dictated by the necessity to account for losses during processing the grain bank flour. Changes were made by consensus at kebele level among HEWs, women's groups, kebele administration, development agents, and women's affairs representatives. At woreda and kebele level, referred to as community level, few respondents elaborated opinions on the bartering model, and in all regions, they generally described the bartering system as “simple” to understand. Caregivers and husbands were equally comfortable with this mode of exchange and appreciated gaining a more nutritious meal in exchange of a single crop. For several caregivers, bartering was the only transaction possible. One mother shared

“It will be difficult for us to buy the complementary food from the market, since it requires some cash payment. In the current arrangement, what we contribute is what we have at home and our labor, which are both at our disposal. For this reason, the current arrangement is better than accessing the complementary food from the market.”—Participant mother, SNNPR.

However, unavailability of cereals to barter at the household was a barrier for some families to participate, and these respondents preferred cash transactions. Those experiencing drought felt more vulnerable and reluctant to exchange their crop. Supply of agricultural produce followed a seasonal pattern and legumes in general was scarce all year long. The latter presented a challenge for the grain bank to ensure production per the predetermined ratio of legumes and cereals in the complementary food.

In addition to cereals for bartering, producing complementary food at the grain bank required contributions from communities for firewood, milling services, and occasionally transport. For poor families and communities, the lack of financial and material resources was “a major” barrier for the participation in the grain bank scheme. The bartering system therefore questioned the project's sustainability and ability to provide equal access to the grain bank complementary food.

#### 3.2.2 Managing the Grain Bank project

The management features of the intervention were perceived as complex and more challenging than anticipated. First, multiple partners at different levels required additional coordination efforts and time to develop effective collaboration. Second, the intervention was composed of multiple components that needed to be sequenced and coordinated to produce the intervention benefits: construction of grain banks, procurement of agriculture produce, training, production of grain bank flour while ensuring food safety, and finally, distribution and nutrition education. Many respondents reported that a poor planning caused major delays in the implementation of the intervention. Initial delays in construction of the grain banks triggered a domino effect that impacted all subsequent stages of the project. Lack of guidelines and expertise of all the involved parties regarding construction and procurement of raw produce was the main explanation given by stakeholders interviewed. These shortcomings point to insufficient assessment of risks at the conception of the project and consequently an unrealistic timeframe allocated to achieve the expected results in this specific context.

### 3.3 Attributes of the implementers

#### 3.3.1 Human resources at community level

Respondents generally lauded the commitment of the Grain Bank project implementers. At community level, the main implementing actors were the HEWs (from the health sector), the development agents (from the agriculture sector), the NGO coordinators, the volunteers producing the complementary food, and the kebele managers. These individuals were described as champions who invested time, physical effort, and resources for the success of the Grain Bank project despite challenges.

Consistent with the original scaling up strategy, the HEWs had a crucial management role in the project. They were given responsibility for management, monitoring of the production and distribution activities in the grain banks, and mobilizing and organizing the women's groups. Overall, respondents reported that HEWs were highly respected by the community and committed to the project.

However, the HEWs implemented the Grain Bank project activities in addition to their regular duties, and many respondents indicated that this increasing workload affected supervision and monitoring of the grain bank and occasionally caused interruption of complementary food production and distribution. The support received from other actors at kebele level, particularly the development agents and kebele managers, influenced the HEWs' perceived burden. When support was maximal, grain bank activities were performed more satisfactorily. In the project design, the development agents shared a leading role with the HEWs in grain bank management by overseeing the quality of the grains during purchasing, ensuring firewood supply, and community mobilization through their access to farmer families. In the grain banks under study, few development agents were proactive and generally had punctual interventions responding to requests from HEWs.

Attempts to transferring responsibilities from the HEWs to the Health Development Army group failed because of rigid administrative policies assigning each to specific tasks or limited human capacity. In the opinion of some regional coordinators and local stakeholders, most of the Health Development Army group members lacked the capacity or the inclination to take on these responsibilities due to low literacy rates and domestic workload.

Staff turnover, among HEWs, NGO coordinators, development agents, and kebele managers, and lack of training of new staff highly impacted the activities and supervision of the grain bank operations. In most kebeles visited during research, at least one of these local actors had changed during the relatively short lifetime of the project.

#### 3.3.2 Female volunteers

An important group of implementers at kebele level was the female volunteers who produced the complementary food and used their networks^5^ to mobilize women in the community to participate in bartering. In some sites, the women's group consisted exclusively of the Health Development Army leaders, whereas in other sites, there was a mix of Health Development Army and mothers or exclusively mothers of children 6–23 months. Strong organization skills and flexibility in working arrangements distinguished Health Development Army‐exclusive groups from other groups and explained their success in accomplishing their tasks. The women's groups played an important role in scaling up and did not receive incentives. The study revealed several factors that determined the women's motivation to volunteer: social recognition, opportunity to acquire knowledge and skills in preparing complementary food, particularly in mixing cereals and legumes in a 3:1 ratio, and a tradition of women taking joy in working together, as reported in Amhara and Tigray. In Amhara, a Health Development Army leader shares her pride:

“We normally get big appreciation from the community for our participation in this activity and we are happy for that. We have several people who support us to do different activities. They will come when we call them and extend their support. There is also fun among ourselves when we are together to prepare the food and we depart after completing the work with good feeling.”*—*Women's group member/Health Development Army, Amhara.

However, in the opinion of many respondents, including women's group members, lack of incentives withered their motivation towards the project, assessing their time, physical effort, and occasional monetary contributions surpassed their actual benefit from the project.

### 3.4 Delivery strategy

#### 3.4.1 Integrated approach

The Grain Bank project was deliberately integrated into the existing health system. The process evaluation showed mutual reinforcement between the grain bank activities and the health sector activities. Woreda health staff and HEWs in Amhara and Oromia sites reported better use of the health services by community members since the roll out of the project, as the grain bank offered opportunities for discussion and a meeting venue. A woreda health centre staff member explains

“The Grain Bank project is very helpful for us and facilitates our work since we are using the grain banks at kebele level for the demonstration of complementary food preparation for mothers. Therefore, it minimizes the burden and cost for us in our effort for promotion of proper child feeding practice. (…). Therefore, for us the grain bank has become an indispensable mechanism to achieve our community‐based nutritional objectives.”—Woreda health staff, Amhara.

Respondents from national and regional levels reported that integration was well achieved, whereas respondents at community level underlined the integration challenges caused by human resource limitations.

#### 3.4.2 Cascade training approach

The project used a four‐step cascade training approach: (a) training for the NGO partners at inception of the program, (b) three‐day training of trainers for HEWs and stakeholders from kebele and woreda levels by NGO partners, Addis Ababa University, and regional universities,^6^ (c) one‐day workshop with women's groups in each kebele, and (d) training of other women on complementary food preparation in the community on an ongoing basis by HEWs and Health Development Army.

Many stakeholders perceived knowledge transfer and capacity building as the main goal of the project. Training and practical demonstrations of food preparation to mothers in the community were appreciated for building confidence and skills, acquisition of new knowledge in mixing cereals and legumes in a ratio, and dismissing food taboos such as the benefits of adding kidney beans in complementary food, a pulse disregarded by some local communities. More than the grain bank building or the food distributed, the knowledge and skills gained were praised by mothers, women's groups, and health providers alike. Many mothers and their families showed readiness and even preference to make complementary food on their own at home. One of the participant mothers reported

“We learn how to prepare the complementary food for our children using a mix of maize and haricot bean. Previously we used these food items separately in preparing the food. We got a lot of knowledge from this project. Now we can prepare complementary food on our own, using different food grains and ingredients.”—Participant mother, SNNPR.

However, delayed training caused a loss of momentum, and there was a perceived lack of refresher training. The latter combined with high staff turnover affected the quality of the intervention, in the opinion of some respondents.

### 3.5 Adopting community

The adopting communities consisted of the caregivers of children 6–23 months, both the mothers and the fathers, the kebele management, community, and religious leaders. The intervention used a participatory approach to encourage communities to adopt the Grain Bank project and foster ownership. The approach entailed community involvement in decision‐making and shared responsibility from the inception of the project. Regular community meetings ensured that the grain bank activities were discussed by local authorities and beneficiaries and that technical, managerial, or resource difficulties found home‐grown solutions.

The participatory approach was perceived as a novelty by the communities and highly appreciated at senior and community levels. Communities expressed a strong sense of ownership towards the project:

“We believe the grain bank activity is our own and it is beneficial to us. Therefore, the women group organization looks after it. I think it is the property of the community even though there is an NGO which supported us for its establishment.”—Health Development Army leader, SNNPR.

Congruent with their sense of ownership, some women's groups initiated reflections on sustaining the project without external support, and a few government representatives at community level demonstrated willingness to adopt the project.

Examples of solidarity, such as a school administration securing crops during the abundant seasons for vulnerable families to barter or a self‐help group, “ekub,”^7^ organized by mothers to assist other women in participating in the bartering system, were evidence that efforts to provide an enabling environment had been underway during the program.

Mothers and fathers valued the skills gained from the intervention. Although some fathers appreciated the time saved by centrally producing complementary food, many expressed their opposition to seeing their wives spending a substantial amount of time outside the house diverting their attention from household and other social activities without compensation.

Despite perceived benefits to their children's health, many mothers and their families remained unenthused by the bartering. Some openly expressed their resistance to a project that would not provide free portions of complementary food, perceived to be the norm for NGOs working in the community. Yet, national and regional stakeholders shared the opinion that beneficiaries needed more time to adopt projects requiring substantial contributions from them. One of them shared

“Even mothers with normal economic status insist for free distribution of the complementary food. They say if it is a support to the community, the government shall distribute it for free.”—Health Development Army, Amhara.

### 3.6 Research and monitoring and evaluation

Research and evaluation were planned in the design of the project by national level implementers and donor agencies. However, in practice, this was not fully carried out due to delays in implementation. A baseline study of nutritional indicators was conducted, but midterm and end‐line evaluations to determine the intervention's impact on growth was considered inappropriate given the shorter than anticipated duration of the project.

In many grain banks, including the eight in the study, monitoring reports were incomplete as activities were not consistently registered. A weakness in the monitoring activities was recognized by most implementers. Stakeholders mentioned the high workload of HEWs and NGO coordinators, staff turnover, difficult physical access to remote sites, lack of interest towards reporting by field staff, and the redundancy of the reporting formats, as the main reasons for inconsistent monitoring.

## 4 DISCUSSION

Strong political support, integration into the community health system, and the participatory approach were the main drivers of the scaling up of the Grain Bank project. However, complex management systems, human resource limitations, and sustainability of the project during times of food insecurity were the main challenges encountered.

### 4.1 Political support and integration in the existing health system

Research findings on scaling up health interventions demonstrate the value of aligning projects to national priorities and integrating them into existing health structures (Baker, Sanghvi, Hajeebhoy, & Hailu Abrha, 2013; George, Menotti, Rivera, & Marsh, 2011; Milat et al., 2015; Robert et al., 2006). In its design, the Grain Bank project recognized and included these critical elements. The Ethiopian government clearly identified child undernutrition as a priority for the country (Federal Democratic Republic of Ethiopia, 2009) and the Grain Bank project benefitted from a favourable political environment to gain government buy‐in at national and local levels. Thus, integrating the project into the kebele health care system was successfully implemented, despite some enduring challenges.

First, integration of the project into the health care system was limited to assigning health staff to include grain bank activities into their routine services. Reporting and management systems of the grain bank were not integrated in the local health system procedures. This would have contributed to intervention adoption but demanded deeper integration efforts (Atun, de Jongh, Secci, Ohiri, & Adeyi, 2010).

Second, human resource limitations persisted throughout the project lifetime, and no durable solutions were found. At the kebeles, HEWs, development agents, and kebele managers handled heavy workloads dividing their time among competing priorities. Development agents' and HEWs' high workload have often been reported and was confirmed in our evaluation. Lack of financial and material resources, burden of administrative tasks, and deficient training were also identified as barriers in these studies (Bantayerga, 2011; Teklehaimanot & Teklehaimanot, 2013; Wakabi, 2008).

To mitigate health worker shortages and keep front line health workers motivated, proposed strategies include task shifting, increased training, continuous learning, and creating financial as well as nonfinancial incentives (Bantayerga, 2011; Chen et al., 2004).

For volunteers, the lack of incentives was widely reported as a barrier to the project. This was witnessed in other projects in Ethiopia where lack of incentives was also stressed as inhibiting participants' motivation (Baker et al., 2013; Haile, Yemane, & Gebresselasie, 2014; Kim et al., 2015; Maes, Closser, Vorel, & Tesfaye, 2015).

### 4.2 Participatory approach

The participatory approach was perceived as the true innovation of the project. While a motivator for many, it also hindered involvement for some. This was mainly caused by the required time commitment to fully engage in grain bank activities. Participatory approaches have been proposed by many public health practitioners as a means of ensuring sustainability and effectiveness of development interventions by empowering the targeted community (Cleaver, 1999). There is some evidence that efforts which involve beneficiaries from the start are more effective than those which do not (Rifkin, Lewando‐Hundt, & Draper, 2000). In our study, this approach empowered the community, fostered a sense of ownership, and reinforced coping strategies. However, it did not always materialize into an increased mobilization of mothers to participate in the bartering. As noted by Rifkin, Hewitt, and Draper (2007) and stated by many respondents in the study, adoption of innovations requires time. The relatively short timeframe of the intervention may have been insufficient to overcome initial resistance to change.

### 4.3 Sustainability of the bartering system

Central to the intervention, the bartering model raised concerns over its sustainability, particularly in a context of community or household food insecurity. In the pilot Grain Bank project, agriculture resource limitations were barriers for mothers to participate in bartering (Roche et al., 2017). Reinforcing the involvement of the agriculture sector in the scaled‐up Grain Bank project intended to overcome this challenge. However, from the results of this study, such barriers to bartering remained for the poorest families. Drought combined with seasonal variations in food availability further restricted mothers' capacity to contribute cereals. Therefore, nutrition interventions that require food contributions should account for seasonality in their design, by assessing them (Wijesinha‐Bettoni, kennedy, Dirorimwe, & Muehlhoff, 2013), setting realistic expectations of the contributions families can make, and planning strategies to overcome the difficulties that may be encountered. Such strategies could include conditional cash transfers during periods of food shortages or temporary food aid to vulnerable families (Bailey & Hedlund, 2012).

### 4.4 Management structures

On management level, scaling up was undermined by complex management structures and limited monitoring of the grain bank activities. Through research conducted in Ethiopia, Bangladesh, and Vietnam, Baker et al. (2013) noted that thorough early preparation, regular monitoring, coordination mechanisms, and continued support to solve operational difficulties are essential for scaling up IYCF programs. Furthermore, Milat et al. (2015) single out M&E systems and costing of interventions as the most essential elements for the success of scaling up. These were reported as deficient in the Grain Bank project. Nonsystematic use of evidence and weak monitoring prevented course correction at earlier stages of the scale up. Several well known challenges of M&E systems in low resource settings were also identified in this study, such as technical limitations, human resource constraints, and lack of appreciation of the importance of program monitoring and its consequences (Karim et al., 2002; Kusek & Rist, 2004; Nash et al., 2009; The World Bank & Inter‐American Development Bank, 2010). Increased training and ongoing supervision and feedback have shown to improve quality of reporting (Mpofu et al., 2014; Nash et al., 2009). However, additional research is needed to provide guidelines on how to develop and foster local commitment and ownership towards M&E efforts.

### 4.5 Strengths and limitations of the study

The process evaluation was a case study, a methodology chosen deliberately to gain in‐depth understanding of the scaling up process, and to present the experiences and views of key stakeholders involved in the project. The framework guiding our analysis of the scaled‐up intervention enabled understanding of key elements that influenced the process and their interrelations. The wide selection of respondents, the inclusion of poor and well‐performing sites, and triangulation of data sources have contributed to reaching this goal.

However, the selection process for FGDs relied on the active involvement of HEWs and despite efforts from the data collection team to ensure balanced selection of respondents, there is a chance of selection bias. In addition, an unexpected language barrier was experienced during one FGD that obliged data collectors to resort to the HEW as a translator. Therefore, her presence may have oriented the opinions shared by the group.

## 5 CONCLUSION

Scaling up nutrition education by teaching mothers how to produce a more nutritious complementary food was successfully implemented and adopted by communities. Conversely, implementing the grain bank as a unit for production and distribution of complementary food was not uniformly adopted throughout the intervention sites.

Through this case study, the Grain Bank project provides many lessons. First, project timeframe is critical. Sufficient time must be dedicated to build strong management structures and coordination between partners on one hand and to allow communities to learn and adopt innovations on the other. Second, it is essential to ensure integration in existing health structures while mitigating staff shortage and heavier workload. Third, participatory approaches are important but must be commensurate with the communities' actual capacity to contribute. Fourth, in drought prone areas, interventions that require food contributions must build in measures to overcome temporary food shortages.

Above all, the Grain Bank project highlighted the readiness and potential of mothers to learn new skills to improve their child's nutrition. The exchange of new knowledge was the highest valued asset of this program. Empowering dedicated mothers with education on how to improve their child's nutrition should therefore be considered as a key component of scaled‐up IYCF intervention programs.

## CONFLICTS OF INTEREST

The authors declare that they have no conflicts of interest.

## CONTRIBUTIONS

BS, JL, SO, IB, and ATB designed study protocol; BS conducted analysis and interpretation of data and drafted paper; JL drafted the concept of the paper; and AL, JL, IB, AH, SO, and ATB reviewed the paper. All authors read and approved the paper.

## ENDNOTES

1 The smallest administrative unit in Ethiopia.

2 Recipes were developed by Addis Ababa University and regional universities using common local legumes, cereals, and sometimes sugar in a set ratio of 3 parts cereals for 1‐part legumes. Recipes varied per region and woreda.

3 Mothers would contribute two measures of a given cereal and would receive three measures of complementary food containing cereals and legumes.

4 District composed of several kebeles.

5 One to five networks: Each HDA leader is a model for five other women in the community.

6 Hawassa University, Haremaya University, Bahir Dar University, Mekelle University.

7 Traditional system of rotating loans, whereby a group of mothers contribute a small amount every month and one mother receives the total amount each time.
